# Alerting attention is sufficient to induce a phase-dependent behavior that can be predicted by frontal EEG

**DOI:** 10.3389/fnbeh.2023.1176865

**Published:** 2023-05-24

**Authors:** Georgios Mentzelopoulos, Nicolette Driscoll, Sneha Shankar, Brian Kim, Ryan Rich, Guadalupe Fernandez-Nunez, Harrison Stoll, Brian Erickson, John Dominic Medaglia, Flavia Vitale

**Affiliations:** ^1^Department of Bioengineering, University of Pennsylvania, Philadelphia, PA, United States; ^2^Center for Neuroengineering and Therapeutics, University of Pennsylvania, Philadelphia, PA, United States; ^3^Center for Neurotrauma, Neurodegeneration, and Restoration, Corporal Michael J. Crescenz Veterans Affairs Medical Center, Philadelphia, PA, United States; ^4^Department of Psychological and Brain Sciences, Drexel University, Philadelphia, PA, United States; ^5^Department of Neurology, University of Pennsylvania, Philadelphia, PA, United States; ^6^Department of Neurology, Drexel University, Philadelphia, PA, United States; ^7^Department of Physical Medicine and Rehabilitation, University of Pennsylvania, Philadelphia, PA, United States

**Keywords:** rhythmic attention, phase-dependent behavior, theta oscillations, high density electroencephalography, gel-free electroencephalography, biomedical applications of MXene

## Abstract

Recent studies suggest that attention is rhythmic. Whether that rhythmicity can be explained by the phase of ongoing neural oscillations, however, is still debated. We contemplate that a step toward untangling the relationship between attention and phase stems from employing simple behavioral tasks that isolate attention from other cognitive functions (perception/decision-making) and by localized monitoring of neural activity with high spatiotemporal resolution over the brain regions associated with the attentional network. In this study, we investigated whether the phase of electroencephalography (EEG) oscillations predicts alerting attention. We isolated the alerting mechanism of attention using the Psychomotor Vigilance Task, which does not involve a perceptual component, and collected high resolution EEG using novel high-density dry EEG arrays at the frontal region of the scalp. We identified that alerting attention alone is sufficient to induce a phase-dependent modulation of behavior at EEG frequencies of 3, 6, and 8 Hz throughout the frontal region, and we quantified the phase that predicts the high and low attention states in our cohort. Our findings disambiguate the relationship between EEG phase and alerting attention.

## 1. Introduction

At any given time, the brain receives vast amounts of environmental stimuli. To compensate for its capacity limit, the brain has evolved mechanisms to direct its cognitive resources. Among these resources, attention is fundamental to selecting and filtering information (VanRullen, 2018). However, sustaining attention for long periods is challenging, and lapses in attention can have severe consequences. Unfocused surgeons make medical errors, inattentive security guards overlook theft, and distracted pilots can cause accidents (Clayton et al., 2015). It is therefore imperative to understand the neural mechanisms of attention in order to better diagnose non-normative attention issues, and ultimately reverse or prevent them.

Attention has historically been perceived as operating at a constant level over time. Under this conceptualization, deploying attention to one stimulus would result in a quasi-constant level of vigilance to that stimulus. Contrary to this view, evidence supports the notion that sustained attention rapidly cycles between “high” and “low” states (Walsh, 1952; Lansing et al., 1959; Callaway, 1962; Dustman and Beck, 1965; Lakatos et al., 2008; Busch et al., 2009; Mathewson et al., 2009; Fiebelkorn et al., 2013, 2018; Helfrich et al., 2018; VanRullen, 2018). “High” refers to states where attention is more efficient, leading to optimal behavioral performance, while “low” refers to states where attention is less efficient and behavioral performance is sub-optimal or in a transitory mode. This rhythmic cycling of attention has been linked to similarly rhythmic neural activity in the brain (VanRullen, 2016, 2018). Results from multiple independent electroencephalography (EEG) studies involving attention suggest that a relationship exists between attention performance and moment-to-moment neural activity in the *θ* and α frequency ranges (5–15 Hz) (Busch et al., 2009; Busch and VanRullen, 2010; Drewes and VanRullen, 2011; Dugue et al., 2011; Chakravarthi and VanRullen, 2012; Dugué et al., 2015; McLelland et al., 2016; Sherman et al., 2016; Plöchl et al., 2021). Additional studies investigating the mechanisms of visuo-spatial attention invasively with stereoencephalography (sEEG) and electrocorticography (ECoG) also identified periodic fluctuations of behavior whose origin could be traced to the instantaneous phase of ongoing neural oscillations (Fiebelkorn et al., 2018; Helfrich et al., 2018).

Despite substantial evidence suggesting that attention is periodically modulated by ongoing neural oscillations, whether attention (and cognition in general) is intrinsically rhythmic is still unclear (Keitel et al., 2022). The range of frequencies that have been observed to modulate attention are broad (5-15 Hz), and have been shown to depend on task and stimulus characteristics (Ho et al., 2017; Ronconi et al., 2017; Chen et al., 2020; Merholz et al., 2022). Additionally, relationships between attention and phase have been observed in a wide range of brain regions (i.e. frontocentral, occipital, and parietal areas), despite the attentional network having been structurally associated with the frontal and parietal regions of the brain (Fan et al., 2005; Buschman and Kastner, 2015). The limited number of replication studies that have been published, and the inability to replicate previous results also cast doubt to the notion of phase-dependent attention. For instance, van der Werf et al. (2021) was unable to replicate the results of Helfrich et al. (2018) and Vigué-Guix et al. (2020) was unable to replicate the results of Callaway (1962). Furthermore, evaluation of 23 studies that tested rhythicities in cognition summarized by Keitel et al. (2022), resulted in a split picture with 11 studies reporting evidence of rhythicities while 12 studies reported null or inconclusive findings.

We contemplate that a step toward disentangling the relationship between attention and phase comes from using simple behavioral tasks that separate attention from other higher order cognitive functions (such as decision-making and/or perception) and also by the use of hardware that enables high spatiotemporal resolution over the brain regions associated with the attention network. Traditional EEG used in many studies (Busch et al., 2009; Busch and VanRullen, 2010; Drewes and VanRullen, 2011; Dugue et al., 2011; Chakravarthi and VanRullen, 2012; Dugué et al., 2015; McLelland et al., 2016; Sherman et al., 2016) has typically been limited to low density montages. Studies adopting invasive sEEG (Helfrich et al., 2018) and ECoG (Fiebelkorn et al., 2018; Helfrich et al., 2018) mapping have the advantage of attaining excellent spatiotemporal resolution, but are similarly limited by the nature of the tasks (e.g., involving visuospatial attention and perception working concurrently). Furthermore, interpretation of their outcomes is complicated by the underlying neurological abnormalities in the participants (most commonly epilepsy).

In this study, we hypothesized that the phasic information of neural oscillations is related to the capacity to maintain a state of alert arousal, or alerting attention (Fan et al., 2002). To test this hypothesis, we investigated whether the instantaneous phase of ongoing neural oscillations predicts alertness in healthy participants. We focused on alerting attention due to its fundamental nature; it is a required precursor to more complex attention functions, such as orienting and conflict resolution (Galvao-Carmona et al., 2014). We isolated alerting attention using a classic Psychomotor Vigilance Task (PVT) (Dinges and Powell, 1985), in a paradigm similar to that used by Callaway (1962) and Vigué-Guix et al. (2020) to eliminate perception from a potential confounding factor, and attained high spatiotemporal resolution recording of brain activity by using custom high-density dry EEG arrays that have recently been developed and validated (Driscoll et al., 2021). The EEG arrays utilize Ti_3_C_2_T_x_ MXene (Alhabeb et al., 2017; Mathis et al., 2020), a 2D nanomaterial with excellent electromagnetic properties, to enable gel-free EEG recording with very high sensor density and low skin-electrode impedance (Murphy et al., 2020; Driscoll et al., 2021). Contrary to Callaway (1962) and Vigué-Guix et al. (2020), we recorded high-density EEG from bifrontal sites [F3 and F4 (Jurcak et al., 2007)] based on prior evidence suggesting that the attention network is structurally located in this region (Fan et al., 2005; Buschman and Kastner, 2015).

We hypothesized that the participant response time - which is a proxy for alertness - could be predicted by the phase of EEG oscillations and we performed follow-up exploratory analyses (i) to investigate whether this effect was lateralized, (ii) to identify which phase corresponds to high and low alerting attention states. We conducted our analyses at within- as well as between-subject level, to ensure that individual response profiles - which are important for informing personalized therapeutic interventions - would not be obscured by averaging techniques.

## 2. Materials and methods

### 2.1. Participants

We recorded EEG data from 36 healthy participants who gave written informed consent in accordance with the Institutional Review Board of Drexel University (protocol No. 1904007140). After stringent exclusions for quality control described in Section 2.5, we retained 15 subjects for analyses (20.26 years ± 1.28, mean ± SD; 5 female). Each participant was fitted with two 4x4 arrays (16 electrodes/array, diameter: 3mm, height: 5mm, pitch: 6mm, area: 21x21 mm^2^ per array) centered approximately at the F3 and F4 locations of the international 10-20 EEG system (Jurcak et al., 2007), which were located by measuring 12 mm anterior and 3 mm left and right of the C_*Z*_ position. Adjustments were made to place the array below the hairline if necessary. Participants were included in the study if they (i) had no history of neurological or psychiatric illness in the past two years, (ii) had not abused drugs or alcohol within the past year, (iii) were not currently taking any psychoaffective medications. Participants were compensated $25 per hour for their time.

### 2.2. Fabrication of the high-density dry EEG arrays

Dry EEG arrays were fabricated following previously published protocols (Driscoll et al., 2021). Briefly, a nonwoven, hydroentangled cellulose-polyester blend substrate was patterned with a CO_2_ laser into the desired electrode array geometry (Supplementary Figure S1). Next, we infused the cellulose-polyester substrate with a Ti_3_C_2_T_x_ MXene dispersion at 20 mg/mL obtained from Murata Manufacturing Co. (Kyoto, Japan). The ink quickly wicked into the absorbent substrate, coating all the fibers to form a conductive composite. To fabricate 3D mini-pillars, we deposited Ti_3_C_2_T_x_-infused cellulose aerogels cut to shape onto the electrode locations. The arrays were then thoroughly dried in a vacuum oven for 1 h at 70°C and 60 mmHg. The resulting structure is a rough, micro-porous conductive composite, with Ti_3_C_2_T_x_ flakes coating the individual fibers in the textile matrix and foam. The arrays were encapsulated in ~ 1 mm-thick layer of polydimethylsiloxane, followed by degassing and curing. To expose the electrode contacts, the mini-pillars were trimmed to a uniform height of 5 mm using a vibratome (Leica Biosystems).

### 2.3. Behavioral task

PVT presentation was performed using PsychoPy 3 v2021.1.4 (Peirce et al., 2019). Participants were comfortably seated ~120 cm away from a 27 inch screen (LG 27GL850, specs here) mounted on a wall in front of them. Refresh rate was set to 144 Hz. Participants were given a keyboard placed on their lap. Room illumination was measured using Dr. meter LX1330B (specs can be found here) to be: (1) 107 lux when facing the monitor at a distance of 120 cm from it and (2) 815 lux when at the same position but facing toward the ceiling. On each trial, a fixation cross (luminance 350 cd/m^2^) appeared at the center of the screen (background luminance 155.76 cd/m^2^). After a variable delay (2–11 s), a supra-threshold target stimulus (a red dot, luminance 121.12 cd/m^2^) replaced the fixation cross at the center of the screen. The target remained for 2 seconds or until a response was recorded. The target could appear at any point during the delay interval. Participants were instructed to respond to the appearance of the target by pressing the space bar as fast as they could. Participants performed two 10-minute blocks (approx. 85 trials per block), separated by the performance of two other tasks [Attention Network Task (Galvao-Carmona et al., 2014) and N-back (Kirchner, 1958)]. The experimenters monitored the participants via a camera to ensure they maintained fixation and remained alert and engaged with the task.

### 2.4. EEG and behavioral data acquisition

EEG and behavioral data were acquired using the Intan RHD system (Intan RHD2000, Intan Technogies) and digitized at a rate of 2 kHz. EEG was bandpass-filtered online at 0.1–1,000 Hz with the built-in analog filter. The right and left mastoids were used as ground and reference, respectively. Behavioral data were time-synced with the EEG via a TTL pulse sent to the Intan at the time a behavioral event occurred (Fixation, Target, or Response). The event type was encoded by the duration of the TTL pulse.

Since triggering the behavioral events is subject to hardware lag, we also placed a photodiode at the screen where behavioral cues appeared (Fixation, Cue) to monitor the exact time that the stimuli appeared at the screen. The voltage changes of the photodiode signifying the appearance of the fixation cross or the target were also fed into the same EEG recording amplifier. To quantify the time lag between the appearance of the target on the screen and time it took for TTL pulse to encode that event in the EEG recording amplifier, we computed the time difference between *t*_0_: the time that a voltage change that signified the appearance of the target on the screen was detected by the photodiode, and *t*_1_: the time that a TTL pulse was sent to the amplifier to encode the same behavioral event. We performed this procedure for 351 trials while subjects S3 and S4 performed the behavioral task. We identified that the time lag between the appearance of the target on the screen *t*_0_ and the time that the appearance was encoded by the TTL *t*_1_ was Δ*t* = 18.64 ± 5.31 ms (mean ± SD, see Supplementary Figure S2).

### 2.5. EEG and behavioral data preprocessing

EEG data were imported in MATLAB R2021b (Mathworks, Natick, Massachusetts) using a custom script and were populated in an EEGLab (Delorme and Makeig, 2004) compatible structure. Bad channels were detected and excluded from the analysis using EEGLab “clean_artifacts” function recursively until no bad channels were returned. Blinks were identified using Blinker (Kleifges et al., 2017) with the default parameters (stdThreshold = 1.5 and pAVRThreshold = 3) and EMG was detected via visual inspection of the EEG timeseries of each participant. Trials for which a blink or EMG occurred near the vicinity of the target (blink: 0.5 sec before or 1.5 sec after the target, EMG: 0.25 sec before or 0.5 sec after the target) were excluded from further analysis. We note that the choice of using a stricter criterion to exclude trials contaminated by blinks as compared to EMG was based on the fact that blinks contain information in the frequency range 1-20 Hz, which is within the range of interest for our study (2–32 Hz), while EMG contains information in frequencies > 30 Hz (Chen et al., 2019), which is mostly outside the range of interest.

We eliminated response time outliers by z-scoring the response times within participants and excluded any trials whose response time had a z-score >3. Participants with <40 trials after preprocessing were excluded from further analysis to ensure all participants had enough trials to obtain a uniform number of response time samples across phase.

Additionally, we linearly detrended the response times of each participant across trials to account for any progressive increase in response times caused by fatigue (Bjørklund, 1992; Langner et al., 2010). As a control analysis, we also reran all analyses without linearly detrending the response times.

### 2.6. Phase-behavior analysis

To test whether the ongoing neural oscillatory activity in the brain significantly predicted response time, we used methodology similar to Helfrich et al. (2018). Only valid trials were used (participant responded after target onset within a 2 s window). We bandpass-filtered the EEG signals aggregated over all experimental blocks (separately for each electrode) in 17 logarithmically spaced bins from 2 to 32 Hz using zero-phase filtering in EEGlab (pop_eegfiltnew with default parameters) to minimize phase-distortion and avoid edge artifacts (Supplementary Figure S3A).

After filtering, we applied a Hilbert transform to extract the instantaneous phase angles (Supplementary Figure S3B), then binned them at target onset into 50 equally distributed bins, whose centers are contained in the set,


(1) $$\begin{array}{l} S=\left\{-\frac{49\pi }{50}+\frac{\pi }{25}n\;|\;n=0,\;1,\;2,\;\ldots ,\;49\right\}, \end{array}$$

and computed the average response time for each frequency band and for each electrode across all trials within a 90 degree window,


(2) $$\begin{array}{l} W_{s}=\left[s-\frac{\pi }{4},s+\frac{\pi }{4}\right], \end{array}$$

where *s* ∈ *S* is the center of each phase bin (Supplementary Figure S3C). We then calculated the Kullback-Leibler divergence (KL divergence) of the observed distribution *Q* against the uniform distribution *P* (Supplementary Figure S3D) using the formula,


(3) $$\begin{array}{l} D_{KL}\left(P,Q\right)=\underset{x\in S}{\sum }P\left(x\right)\log\frac{P\left(x\right)}{Q\left(x\right)}, \end{array}$$

to quantify how strongly the observed distribution deviated from uniformity.

Statistical significance of non-uniformity was quantified as follows: for each frequency band of each electrode of each subject, we shuffled the observed phase bin - response time pairs 1,000 times and computed the KL divergence *D*_*KL*_(*P, Q*_*perm*_) of each shuffled distribution *Q*_*perm*_ against the uniform distribution *P* using Equation 3 (Supplementary Figures S3C–F). This allowed us to obtain a surrogate distribution of KL divergence values, *D*_*surr*_, for each frequency band of each electrode of each participant. The KL divergence of the observed phase bin - response time pairs was placed in the surrogate distribution and z-scored (Supplementary Figure S3G) using the formula,


(4) $$\begin{array}{l} z=\frac{D_{KL}\left(P,Q\right)-\mu \left(D_{surr}\right)}{\sigma \left(D_{surr}\right)}, \end{array}$$

where μ(*D*_*surr*_) and σ(*D*_*surr*_) are the mean and the standard deviation of *D*_*surr*_, respectively. This procedure was performed across all electrodes and frequency bands for each participant (Supplementary Figure S3H). A z-score > 2 (*p* < 0.05) was considered significant.

### 2.7. Identification of the frequency band(s) with the strongest phase-behavior effect within and across subjects

In order to identify the frequency band(s) whose phase could predict response response time irrespective of electrode location, within each participant we averaged the KL divergence values *D*_*KL*_(*P, Q*) that were obtained, separately for each frequency band, across electrodes, after zeroing out any non-significant values, since they did not represent any phase dependent modulation of behavior (Supplementary Figure S4A, top). This procedure resulted in one KL divergence value for each frequency band of each participant (Supplementary Figure S4A, bottom).

We then performed a permutation test in which we scrambled the observed KL divergence values *D*_*KL*_(*P, Q*) across frequency bands and electrodes 10,000 times (Supplementary Figure S4B, top). Each time, we computed the mean KL divergence value across all electrodes of that subject, separately for each frequency band (Supplementary Figure S4B, bottom). This procedure generated a surrogate distribution of KL divergence values for each frequency band, within which the observed mean KL divergence value of the same frequency band could be z-scored (Supplementary Figure S4C, top). We then z-scored the observed KL divergence value for each frequency band against the surrogate distribution of KL divergences for that same frequency band (Supplementary Figure S4C, bottom). If the observed z-score was > 2 (*p* < 0.05) that frequency band was considered significant.

To identify the frequency band that showed the strongest phase-dependent modulation of behavior across participants, we repeated the same procedure but averaged the observed KL divergence values across the electrodes of all participants, separately for each frequency band. Correction for multiple comparisons was performed using False Discovery Rate (Benjamini and Hochberg, 1995) across all comparisons performed within this section.

### 2.8. Identification of electrode array that show the strongest phase-behavior effect within and across subjects

To identify which array showed the strongest phase-dependent modulation of response times for each participant, we averaged the normalized KL divergence values across all frequencies and electrodes within each array of each participant, after zeroing out non-significant values. We then obtained a surrogate distribution of normalized KL divergence values by shuffling the normalized KL divergence values across frequencies and channels 10,000 times. Each time, we computed the average KL divergence across all frequencies and channels within each array. We then z-scored the observed normalized KL divergence value for each array against the surrogate normalized KL divergence values of the same array. If the observed z-score was >2, *p* < 0.05, we considered that array significant.

We repeated the same procedure to identify which array showed a higher density of electrodes-frequency band pairs whose phase can predict response time in our cohort, but instead of averaging within participants, we averaged the normalized KL divergence values of each electrode array across all frequencies and participants.

### 2.9. Identification of the phase that predicts fast vs. slow response time

To identify the phase that predicts a fast or a slow response across our cohort, we aggregated the response time - phase angle pairs for each frequency band and electrode across the three groups of participants that we identified to show significant phase-dependent modulation of response times: (i) *θ* band (8/15 participants), (ii) α band (5/15 participants), (iii) β band (2/15) participants. For each group, we then performed the procedure described in Section 2.6 to identify significant electrode locations - frequency band pairs whose phase could predict response times.

We also tested whether the phases that correspond to a fast *vs*. a slow response time were antiphase, separately for the group of *θ* band participants at frequencies 2.7–3.2 Hz, 5.3–6.2 Hz, and for the group of α band participants at frequencies 7.4–8.6 Hz. To do so, for each group, we sorted the phase bins based on their associated response times as follows,


(5) $$\begin{array}{l} RT\left(\theta_{0}\right)<RT\left(\theta_{1}\right)<RT\left(\theta_{2}\right)<\cdots <RT\left(\theta_{49}\right), \end{array}$$

where *RT*(θ_*i*_) corresponds to the response time associated with the phase bin θ_*i*_. We then formed the vector of the absolute differences between the phase bins associated with the fastest *Vs*. slowest response times **v**, with entries,


(6) $$\begin{array}{l} v_{i}=|\theta_{i}-\theta_{49-i}|,\;where\;i=0,\;1,\;2,\;\ldots ,\;24. \end{array}$$

We then tested whether **v** was centered around π rad by performing a V-test using the function “circ_vtest” available in the CircStat toolbox for MATLAB (Berens, 2009).

## 3. Results

**Figure 1:**
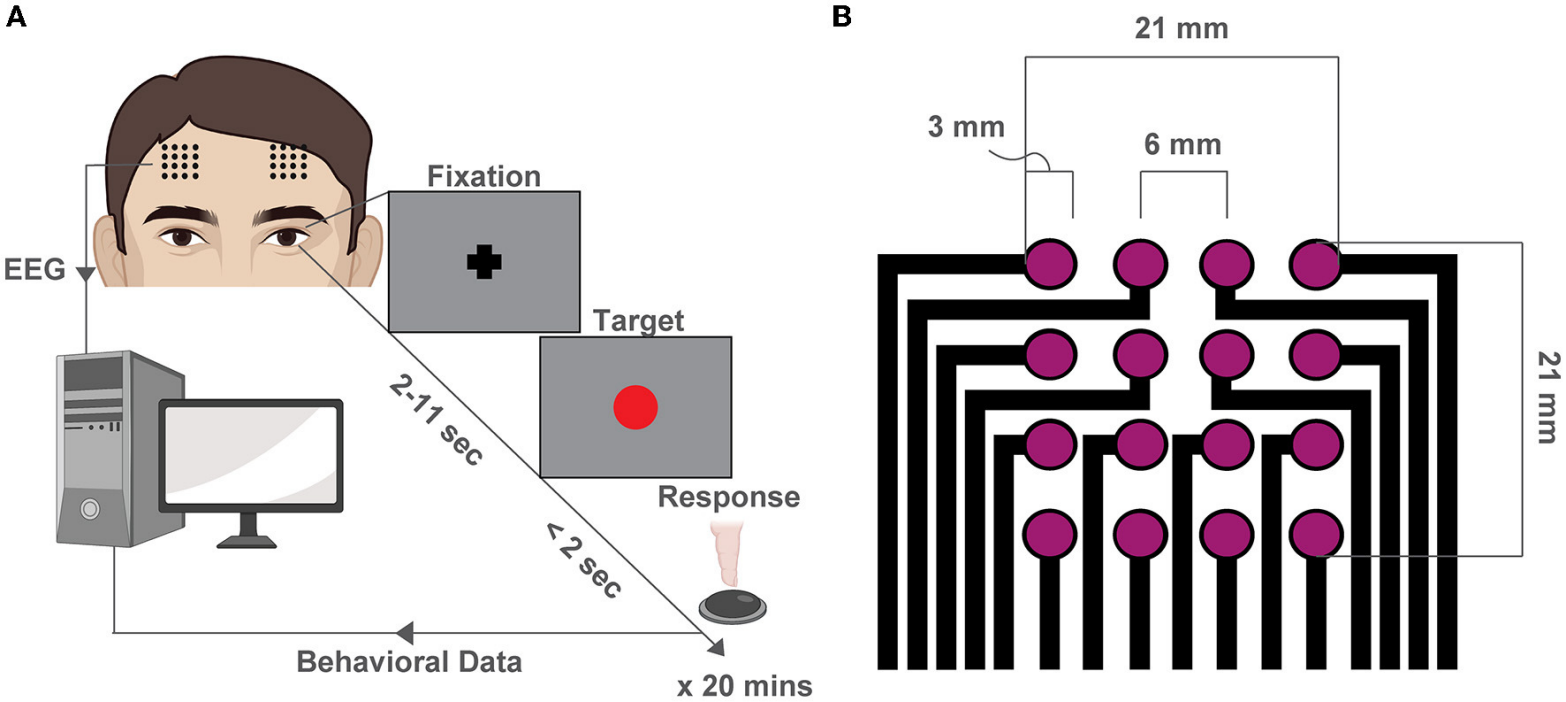
Behavioral experiment and high-density EEG array design. **(A)** Schematic of the PVT. At the beginning of each trial, a fixation cross appears at the center of the screen. After a variable delay (2–11 s), a red dot (the target) fixation cross at the center of the screen and remains there for 2 s or until a button-press response is recorded. The trial then restarts. **(B)** Schematic of the custom high-density array design used to record EEG during the PVT at F3 and F4 locations.

We recruited 36 total subjects and after stringent exclusions for quality control, we retained 15 subjects for the subsequent analyses. The mean number of trials for each participant was 76.7 ± 23.7 (mean ± SD, see Supplementary Table S1). We recorded EEG while participants performed the PVT task (Dinges and Powell, 1985) (Figure 1A) with two 4x4 square grid dry EEG arrays (contact: 3mm diameter, pitch: 6mm, area: 21x21 mm^2^ per array) at the F3 and F4 locations, as specified by the international 10–20 system (Jurcak et al., 2007) (Figure 1B, Supplementary Figure S1).

### 3.1. The phase of EEG predicts alertness

**Figure 2:**
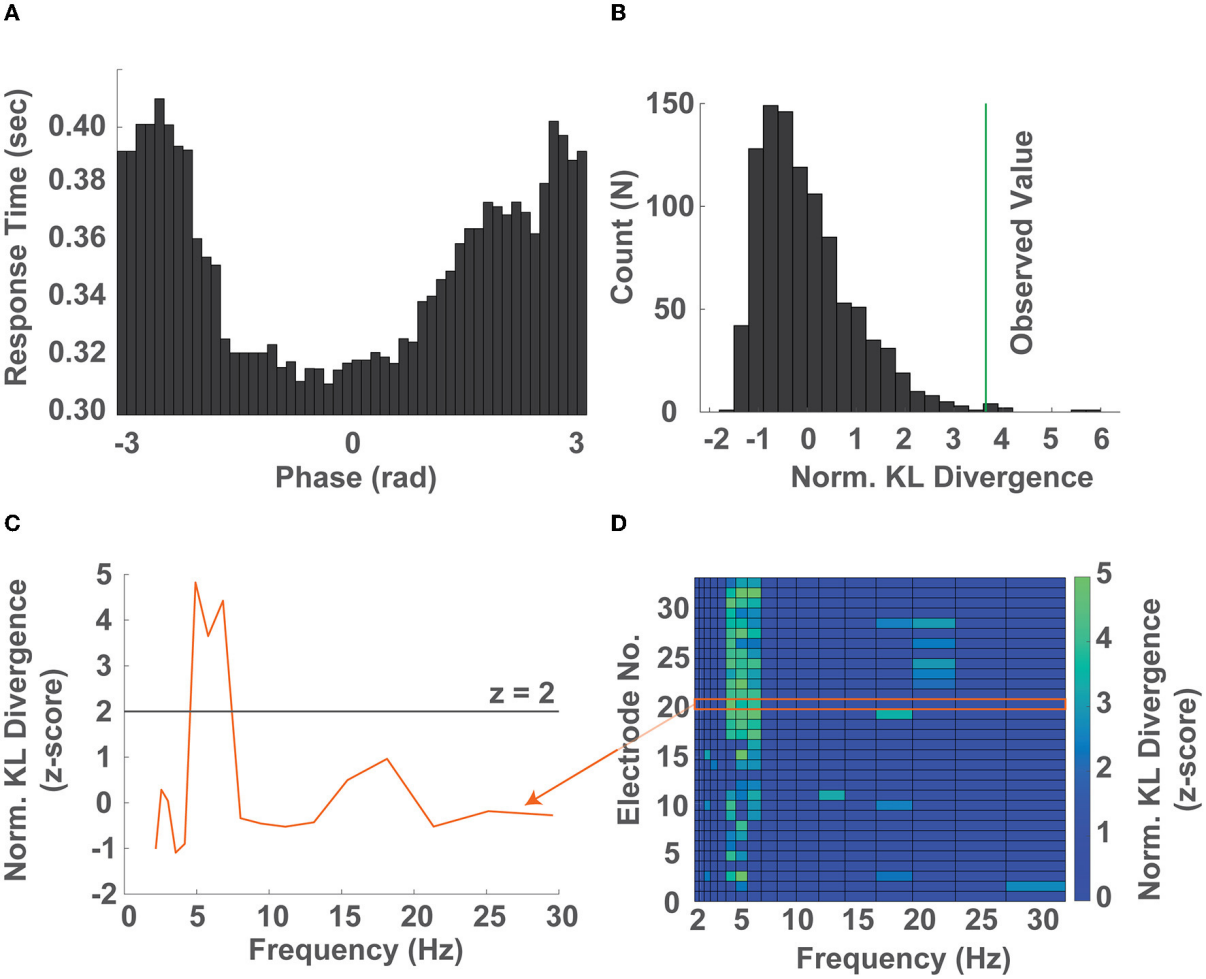
The phase of ongoing neural oscillations predicts response times. **(A)** Distribution of response times as a function of phase across 50 bins (± 45^*o*^) in the 5.3-6.2 Hz band (single electrode). **(B)** Surrogate distribution of KL divergences (gray bars) obtained by randomly shuffling the response time - phase bin pairs and the KL divergence calculated by comparing the observed response time distribution over phase against a uniform distribution (green line) after normalization (single electrode). **(C)** Normalized KL divergence (z-score) obtained by comparing the observed response time over phase distributions against a uniform distribution across all frequency bands from 2-32 Hz. The frequencies whose normalized KL divergence (z-score) exceed 2 were considered significantly non-uniformly distributed response times over phase distributions (single electrode). **(D)** Normalized KL divergence of the observed distributions of response time over phase compared to the surrogate distribution across all electrodes and frequency bands. Non-significant values were set to zero. Elements whose normalized KL divergence is greater than 2 correspond to electrode location - frequency band pairs whose phase significantly predicts the participants response time (all electrodes, representative single participant). Note that the orange arrow connecting the curve of **(C)** and region in **(D)** denotes that the two contain the same information.

In order to investigate the relationship between instantaneous EEG phase and response time, we computed the timecourse of the instantaneous phase of each trial within narrow logarithmically spaced frequency bands spanning 2–32 Hz (Figure 2A). Within each narrow band we sorted the response times into 50 equidistant bins representing the instantaneous EEG phase at target presentation and computed the average response time within each bin (±45^o^). We then quantified how strongly the observed response times were modulated by the phase of EEG activity by comparing their distribution to the uniform distribution using the KL divergence in a permutation test (Figures 2B, C, Supplementary Figure S3). This approach allowed us to identify the frequency band whose phase predicted response time, as well as whether the observed phase-dependent modulation of response time occurred preferentially at particular electrodes (Figure 2D).

We found evidence of phase-dependent modulation of behavior at the frontal region of the brain in 14/15 participants. Interestingly, the frequency band whose phase predicted response time differed across participants, and for some participants, multiple frequency bands exhibited a phase behavior relationship (Supplementary Figure S5). These finding suggest that cortical oscillations in the frontal region of the brain, as measured by EEG, are behaviorally relevant and predict participant alertness at the single subject level.

### 3.2. *θ*-phase shows the strongest behavioral relevance across subjects

**Figure 3:**
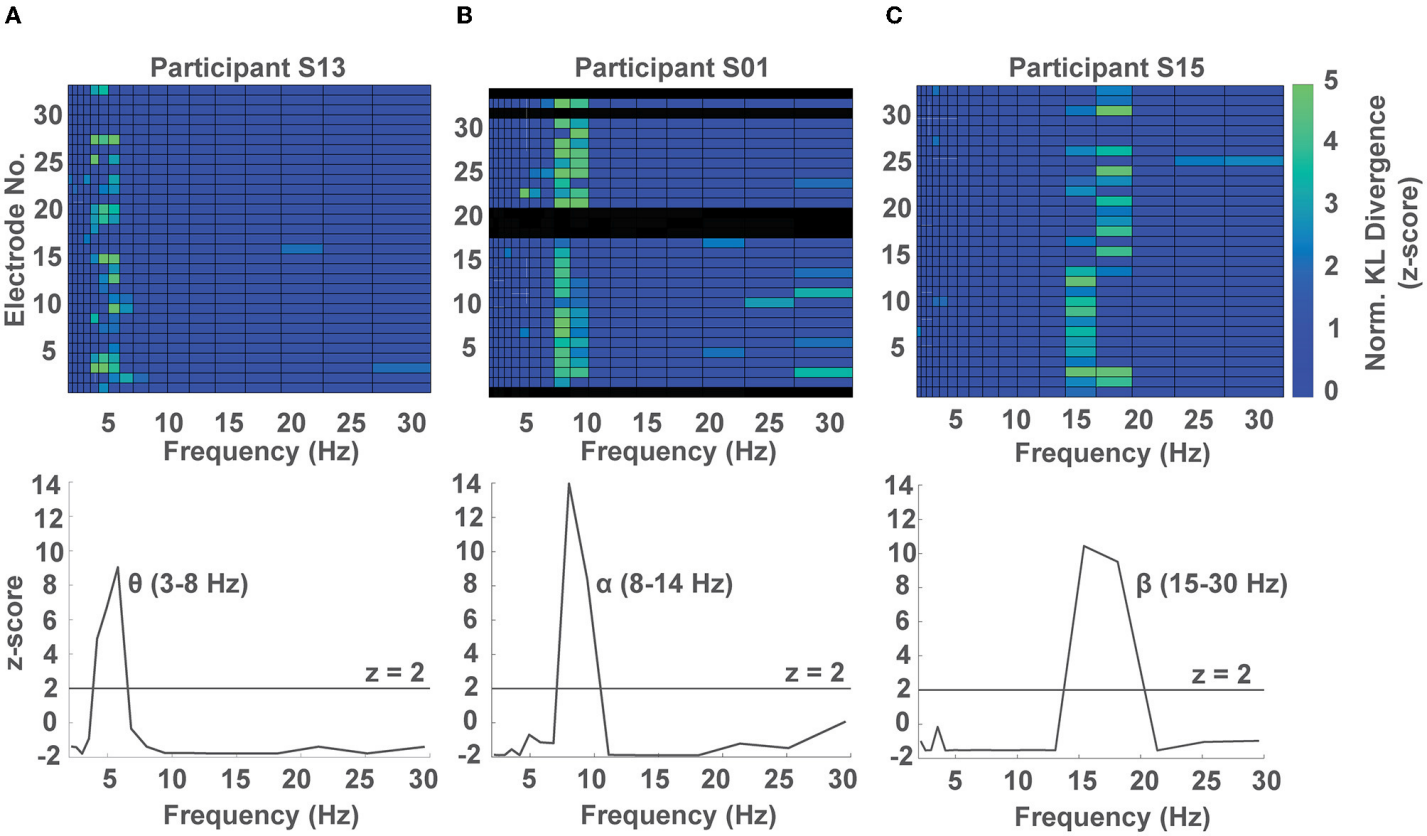
Representative phase-dependent modulation of response at the subject level. **(A–C)** Show a phase-dependent modulation of response times for three participants at different frequency bands. Top: subject-level colormap of the normalized KL divergence at each frequency band by electrode pair (non-significant values were set to 0). Bottom: average normalized KL divergence across channels showing a phase dependent modulation in the θ, α, and β bands for the participants S13, S01, and S15, shown in columns **(A–C)**, respectively. Black rows in the subject-level colormap correspond to high impedance channels.

**Figure 4:**
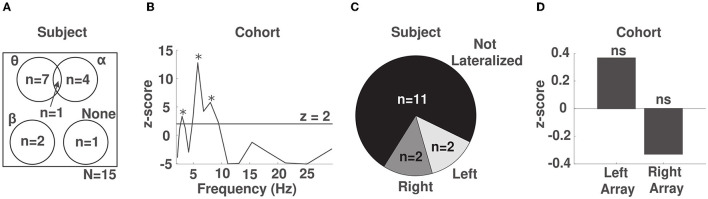
Phase-behavior effects are strongest in the *θ* band across the frontal region. **(A)** Venn diagram of the phase-behavior distribution across frequency bands in our cohort. **(B)** Z-scores obtained by comparing the observed distribution of response times over phase to the uniform distribution using a permutation test across our cohort. Spectral peaks that were significant after correcting for multiple comparisons are marked with an asterisk. **(C)** Distribution of the lateralization of phase-behavior effects within subjects. **(D)** Lateralization effects at the cohort level. ns: not significant.

Having identified multiple frequency band by electrode combinations whose phase predicted response time, we were interested in identifying in which frequency band the phase showed the strongest modulation of response time irrespective of sensor location within each participant. To do so, we computed the average normalized KL divergence at each frequency band across channels within each participant and z-scored the observed values against a surrogate distribution obtained using a permutation test for the same participant (see Methods 2.7, Supplementary Figure S4). In 8/15 participants, we observed a significant phase dependent modulation in the *θ* band (2.5–7 Hz, example participant in Figure 3A), while in 5/15 participants we observed a significant phase-dependent modulation in the α band (8–14 Hz, example participant in Figure 3B), and in 2 participants, we found a significant phase-dependent modulation in the low β band (15–20 Hz, example participant in Figure 3C). In one participant we did not find any phase-dependent behavioral modulation. We note that for 1 participant, phase-dependent modulation of response times occurred in both the α and *θ* bands (Figure 4A, Supplementary Figure S6).

To identify the frequency bands whose phase reliably predicted response time across participants, we pooled the normalized KL divergence values across the electrode channels of all participants, separately for each frequency band, and repeated the same statistical procedure. Across participants, we identified significant spectral peaks indicative of phase-dependent modulation of response time at frequencies 3 (*p* < 0.0006), 6 (*p* < 10^−10^), and 8 (*p* < 10^−8^) Hz (Figure 4B). We note that the spectral peak at 8 Hz was not significant when response times were not detrended to account for fatigue (see Supplementary Figure S7). This suggests that alerting attention alone is sufficient to induce a phase-dependent modulation of behavior in the *θ* band.

### 3.3. Weak evidence for lateralization of phase-behavior relationships

We next explored whether phase-behavior relationships lateralized to the either the right or left array. Within subjects, we computed the average normalized KL divergence across the electrodes and frequencies within each array. We then used a permutation test (see Methods in 2.8) to determine whether the density of identified phase-response time relationships was preferentially distributed to one of the arrays. Within subjects, we observed lateralized effects in 4/15 subjects, 2/15 right-lateralized and 2/15 left-lateralized (Figure 4C). We also investigated whether any lateralization effects were present across subjects in our cohort, after aggregating the data across each array and all subjects. We did not identify lateralization across the cohort (*p* = 0.47 for right hemisphere and *p* = 0.67 for left hemisphere, Figure 4D). This finding suggests that the phase of EEG across the entire frontal region is relevant to alertness.

### 3.4. Identifying the phase of EEG that predicts fast vs. slow response times

**Figure 5:**
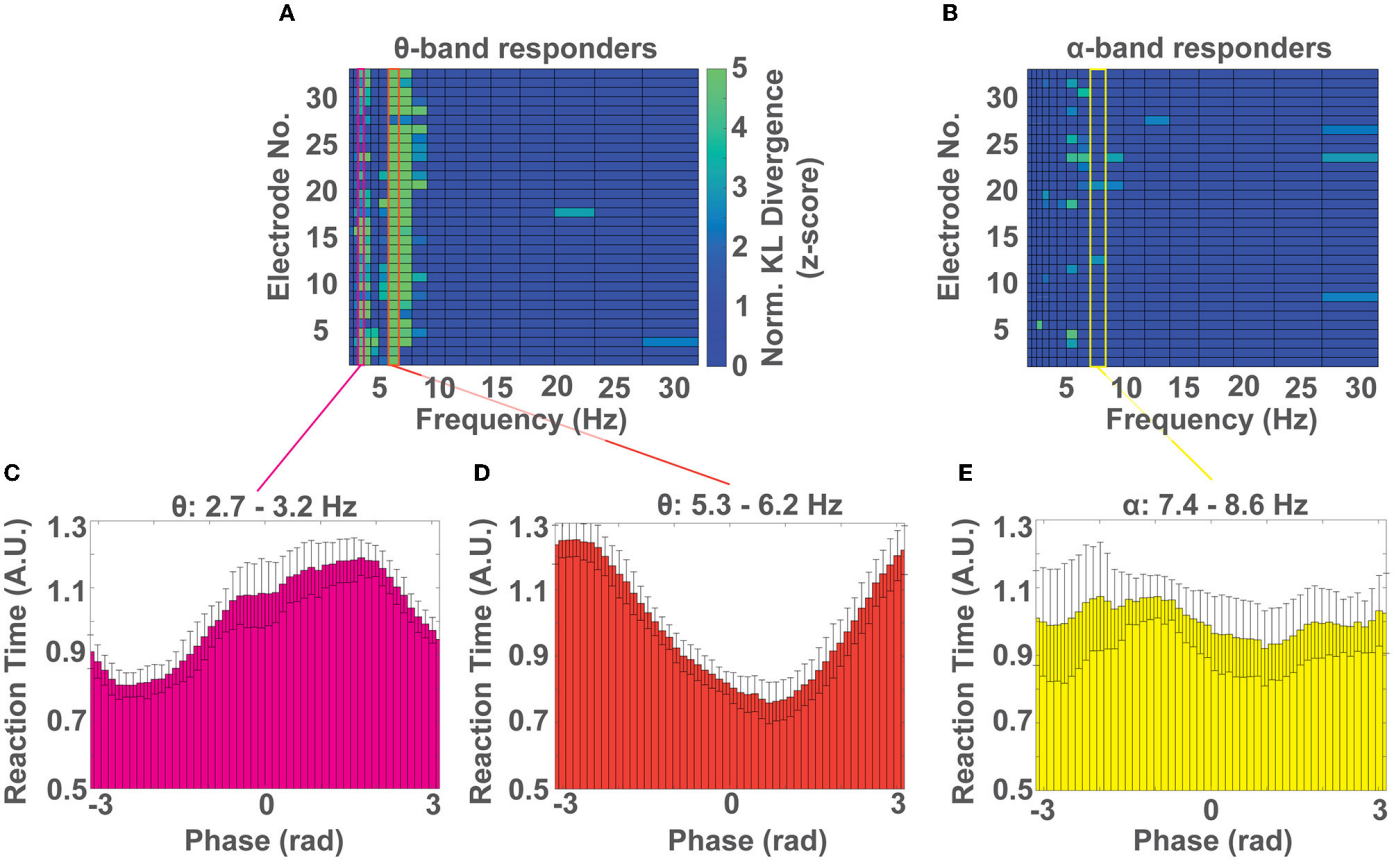
Which phase predicts a fast or slow response time? **(A)** Colormap of normalized KL divergence values obtained by comparing the distributions of response time over phase for all frequency bands and electrodes across all participants showing a phase-dependent modulation of response times in the *θ* band. In this group, the phase-dependent modulation of behavior at frequencies ~3 Hz, and ~6 Hz, can be observed on most channels. **(B)** Colormap of the normalized KL divergence values obtained by comparing the distributions of response time over phase for all frequency bands and electrodes for all participants who showed a phase-dependent modulation of response times in the α band. In this group a non-uniform distribution of response times over phase can be found only on few electrodes. **(C, D)** Distribution of normalized response times over phase for participants with a phase-dependent modulation of response times in the *θ* band at the frequency range ~3 Hz **(C)** and ~6 Hz **(D)**. **(E)** Distribution of normalized response times over phase for participants with a phase-dependent modulation of response time in the α band (~8 Hz). **(C–E)** Show the mean ± SD of distributions across channels.

Next, we investigated which phase of the EEG cycle (i.e., peak, trough, and in-between) was predictive of response time speed (i.e., fast *Vs*. slow) in each of the 3 spectral peaks that showed behavioral relevance (3, 6, and 8 Hz). To do so, we pooled the response time - phase pairs for each frequency band across the participant subgroup that showed phase-dependent modulation of behavior at that frequency and repeated the analysis outlined in Methods 2.6. For the group of *θ* band responders, we identified multiple electrode channels whose distribution of response times over phase were significantly non-uniform (p < 0.05) at frequencies of 3 Hz, and 6 Hz, which aligns well with our cohort level results (see Results 3.2 and Figure 5A). For the group of α band responders, we identified a significant phase dependent modulation of behavior at 8 Hz for few electrodes (Figure 5B). We then averaged the distributions of response time over phase across electrodes for frequencies 3 Hz and 6 Hz for the *θ* band responders and for frequency 8 Hz for the α responders. We identified that the phase that predicts the fastest response time for the *θ* band responders at frequency 3 Hz is centered at $-2.73\pm \frac{\pi }{4}$ rad (Figure 5C) and at 6 Hz is centered at $0.71\pm \frac{\pi }{4}$ rad (Figure 5D). For the α band responders, at frequency 8 Hz the fastest response is centered at $0.96\pm \frac{\pi }{4}$ rad, although the effect was less pronounced (Figure 5E). In all cases, the phase that predicts a slow response time was qualitatively located ~π rad from the phase that predicts a fast response time. We identified this antiphase effect to be significant using a V-test for *θ* band participants at frequencies ~3 (*V* = 10.19, *p* < 0.002) and ~6Hz (*V* = 9.84, *p* < 0.003). The effect was not significant for the α band participants at frequencies 7.4-8.6 Hz (*V* = 3.26, *p*>0.17).

## 4. Discussion

In this study, we demonstrate that alerting attention alone is sufficient to induce a phase-dependent modulation of behavior that can be predicted by frontal EEG. In agreement with some previous studies (VanRullen, 2016, 2018), we identified *θ* oscillations to be the strongest and most consistent predictors of alerting attention. This effect was not lateralized to the right or the left hemisphere as has been the case in some other studies (Busch and VanRullen, 2010; Song et al., 2014; Fiebelkorn et al., 2018; Helfrich et al., 2018). We also observed that the instantaneous phases that predict fast and slow response times across our cohort in the *θ* band are antiphase with each other, further strengthening the relationship between EEG oscillations and alerting attention performance. Since we evoked these phase-behavior relationships using the PVT, which does not involve perceptual judgements, our results support the *θ* phase-dependency theory of alerting attention specifically.

Our findings are consistent with recent evidence suggesting that the nature of sustained attention in humans and non-human primates is rhythmic and depends causally on endogenously generated neuronal oscillations that manifest in the EEG as *θ* (3–8 Hz) (Busch et al., 2009; Chakravarthi and VanRullen, 2012; Fiebelkorn et al., 2013, 2018; Dugué et al., 2015; McLelland et al., 2016; Helfrich et al., 2018) or α band (8-14 Hz) activity (Callaway, 1962; Busch and VanRullen, 2010; Drewes and VanRullen, 2011; Dugue et al., 2011; McLelland et al., 2016; Sherman et al., 2016). It contrasts, however, multiple recent works that failed to identify phase behavior relationships or showed inconclusive findings (Benwell et al., 2017; Ronconi et al., 2017; Rassili and Ordin, 2020; de Graaf and Duecker, 2021; Lin et al., 2021; Michail et al., 2021; Michel et al., 2021; Morrow and Samaha, 2021; Sheldon and Mathewson, 2021; Sun et al., 2021; van Es et al., 2021; London et al., 2022), even when the studies were replicating previous findings (Vigué-Guix et al., 2020; van der Werf et al., 2021).

There is a variety of reasons that could result in inability to identify phase-behavior relationships, such as inadequate statistical power, low signal fidelity, or use of sub-optimal methodologies to detect effects when their magnitude is small (Keitel et al., 2022). We believe that in our work we were able to detect a relationship between EEG phase and attention mainly due to the following reasons. First, we used a simple behavioral task (Dinges and Powell, 1985). Second, we ensured high signal fidelity at the regions of interest using MXtrode EEG (Driscoll et al., 2021) and enforced strict quality control for subjects included in our study. Third, we used methods that are able to accurately detect phase-behavior relationship whenever they are present (see Supplementary Figure S8 and Zoefel et al., 2019).

The PVT used in this study does not rely on saccades, perceptual components, or other higher order attentional functions such as orienting or conflict resolution. To the best of our knowledge, at least one of these functions was explicitly engaged by the tasks used in other works, with the exception of Callaway (1962) and its replication study by Vigué-Guix et al. (2020). Although the behavioral paradigm used by Callaway (1962) and Vigué-Guix et al. (2020) would be sufficient to disentangle alerting attention from higher order cognitive functions, we encourage the reader to interpret their results in the context of attentional processes with caution. The attention network has been structurally associated with the frontal and parietal brain circuits (Fan et al., 2005; Buschman and Kastner, 2015), therefore, it is possible that their findings, which come from occipital electrodes reflect rhythimities in visual processing, in the study of Callaway (1962) and the absence of those in the case of Vigué-Guix et al. (2020). PVT performance is thought to specifically reflect the alerting function of attention, which is necessary to perform most - if not all - more complex attention functions. Because alerting to a stimulus necessarily precedes most other attention functions, it may be commonly activated in complex attention tasks. As such, our ability to specifically isolate the phase-behavior relationships in the EEG with the alerting function, suggests that alerting function alone is sufficient to explain *θ* predictive power in some other studies involving more complex attention tasks. Moreover, we demonstrated that it is possible to identify these signals non-invasively with high-density dry EEG montages placed over the frontal lobes.

Another major implication of this study is the finding that alerting attention might be concurrently modulated by multiple independent rhythms. We conducted analyses at the single-subject and group levels and identified multiple frequencies related to alerting attention (Supplementary Figure S5). Although our within-subject analyses revealed variability in the most behaviorally sensitive frequencies at the individual level, phase-dependent modulation of behavior was most frequently observed in the *θ* band (3-8 Hz), which is consistent with prior studies that performed analyses at the group level rather than within each subject. Across subjects, we identified a periodic modulation of behavior by the *θ* band (3 and 6 Hz) and low α band (8 Hz) activity, although the α band effect was not present when response times were not linearly detrended to account for fatigue (Supplementary Figure S7). Interestingly, the identified spectral peaks were well-separated. VanRullen (2018) proposed that the attentional sampling rhythm in humans is likely at 8 Hz, and further suggested that findings from studies identifying periodicities at lower frequencies such as 3–4 Hz (Landau and Fries, 2012; Fiebelkorn et al., 2013, 2018; Dugue and VanRullen, 2014; Song et al., 2014; Huang et al., 2015; Helfrich et al., 2018) could be a result of divided attention between two locations, focusing at either object during alternate cycles, with the result that periodicity measured at any one location would actually be one half of the 8 Hz intrinsic rhythm of attention. However, our results suggest otherwise. In our task, participants were instructed to fixate at a single location and detect a perceptually suprathreshold stimulus but we still observed two spectral peaks, one at 3 Hz and one at 6 Hz. This suggests that neural oscillations at both frequencies might modulate alerting attention and could possibly serve different functional roles in that context. A similar notion where many simultaneous rhythms periodically modulate a cognitive function at independent rates has been suggested for perception (VanRullen, 2016).

Rhythmicity is likely an intrinsic characteristic of alertness. Previous studies have demonstrated that phase-behavior relationships can be observed in the absence of external rhythms or can be entrained (Busch and VanRullen, 2010; Landau and Fries, 2012; Fiebelkorn et al., 2013). For instance, endogenously generated sampling behaviors such as eye movements in primates (Otero-Millan et al., 2008; Bosman et al., 2009; Hogendoorn, 2016; Wutz et al., 2016) and whisking in rodents (Fanselow and Nicolelis, 1999; Berg and Kleinfeld, 2003) have been attributed to intrinsic neural activity, while external stimuli have also been shown to induce phase behavior relationships in visual attention (Lakatos et al., 2008; Gray et al., 2015). In our study, we removed external oscillatory stimuli from the surrounding environment during data collection and jittered the variable delay of our behavioral task, making entrainment of neural oscillations unlikely to occur. Additionally, the absence of a pre-stimulus cue prior to the target presentation in our behavioral task suggests that the neural state at the time of stimulus onset could impact behavioral outcomes, which implies that rhythmicity is an intrinsic characteristic of the neural mechanism of alerting attention.

Given our results, it is critical to further distinguish the basis of links between fundamental brain frequencies and cognitive processes, and in particular to further evaluate the role of neural processes in attention and perception. Studies investigating attention using different behavioral tasks and electrophysiology such as MEG/EEG (Busch et al., 2009; Busch and VanRullen, 2010; Drewes and VanRullen, 2011; Dugue et al., 2011; Chakravarthi and VanRullen, 2012; Dugué et al., 2015; McLelland et al., 2016; Sherman et al., 2016; Fiebelkorn et al., 2018; Harris et al., 2018; Helfrich et al., 2018; Hauswald et al., 2020; Balestrieri et al., 2021; Ho et al., 2021; Plöchl et al., 2021; Zazio et al., 2021) have suggested that neural oscillations throughout the 5-15 Hz range could be relevant to attention. However, when the results of 9 independent EEG studies involving attention and perception working together were meta-analyzed (VanRullen, 2016), two well separated spectral peaks at frequencies 7 Hz (Busch et al., 2009; Busch and VanRullen, 2010; Chakravarthi and VanRullen, 2012; Dugué et al., 2015; McLelland et al., 2016) and 11 Hz (Drewes and VanRullen, 2011; Dugue et al., 2011; McLelland et al., 2016; Sherman et al., 2016) emerged as the frequencies whose phase was relevant for predicting behavioral outcomes. The peak at 7 Hz was mostly observed in frontal electrodes, and the 11 Hz peak was mostly observed in occipital electrodes. These results suggest that frontal circuits are potentially engaged during attention-linked mechanisms regulating trial-wise behavior, whereas occipital signals represent visuo-perceptual tracking and decoding of stimuli. In this study, we took a focused approach in which electrodes were located in frontal sites only to maximize sampling with our custom high-density arrays (centered on F3 and F4). In close agreement with prior studies, we found the strongest phase behavior relationships at 6 Hz, followed by a weaker peak at 8 Hz in few subjects. Conspicuously when compared to prior studies, our behavioral task (Dinges and Powell, 1985) does not involve perception of a near-threshold stimulus, but rather, a supra-threshold one. Therefore, we speculate that neural oscillations in the α band, close to the 11 Hz spectral peak might be most strongly involved in cued detection in perception or visual processing, while the neural oscillations at frequencies 7 Hz might reflect cognitive processes more closely related to alerting attention specifically (VanRullen, 2016, 2018). A similar notion of *θ* band subserving attention while α band subserving perception was proposed by Michel et al. (2021). This notion is further supported by the fact that the attentional control network has been structurally associated with the frontoparietal region of the brain (Posner and Petersen, 1990; Fan et al., 2002, 2005; Buschman and Kastner, 2015), consistent with our recording locations.

Since the oscillatory phase of distinct frequencies predicted alertness at the individual and group level, one could hypothesize that individual variability in the frequency that modulates alertness might also predict better or worse overall efficiency of alerting attention across subjects. Therefore, we investigated whether the dominant spectral peak frequency that modulated attention within each participant correlated with their average response time across trials. We did not identify evidence to support that notion within our cohort. Due to our small cohort size (*N* = 15) we cannot rule out that our study was underpowered to detect such a relationship (data not shown). Since the mean response time across participants varies, we consider that it is likely that one or more cognitive variables influence the tonic levels of alertness of each participant (Degutis, 2010). Another candidate could be the participant overall fatigue levels, which manifest as a progressive increase in the response time over the course of the task (Bjørklund, 1992; Langner et al., 2010).

This work contributes to a growing literature (Fiebelkorn et al., 2013; VanRullen, 2016, 2018; Helfrich et al., 2018) suggesting the feasibility of phase-guided neuromodulation approaches. The ability to track intrinsic rhythms of basic cognitive functions like alerting attention that gatekeep more sophisticated attention processes could improve the effectiveness of non-invasive closed-loop neuromodulation. Such approaches have been hampered by the limitations of existing hardware that can reliably track brain oscillatory activity non invasively with adequate spatiotemporal precision [with exceptions, see Callaway (1962) and Vigué-Guix et al. (2020)], as well as by a lack of compatibility of those technologies with safe and effective non-invasive neuromodulation, such as transcranial magnetic stimulation (TMS) (Tremblay et al., 2019). Recently developed, dry EEG arrays, such as the MXtrodes, could address those limitations through their customizability in terms of footprint, density, coverage, compatibility with neuroimaging modalities, and highly reliable signal quality (Murphy et al., 2020; Driscoll et al., 2021). We believe that integrating these arrays with existing TMS protocols that have been proven very effective but have not closed the loop yet (Cole et al., 2020) or with EEG/TMS technologies such as in Zrenner et al. (2018, 2020) informed by EEG phase, could further improve outcomes for patients affected by attention-related disorders, such as schizophrenia (Carter et al., 2010), depression (Keller et al., 2019), and generalized anxiety disorders (Yiend et al., 2014) where attentional issues are present (Tremblay et al., 2019).

Some limitations apply to the current study. We found evidence that the phase of neural oscillations can predict response times during the PVT. However, we did not evaluate rhythmicity in our behavioral data, such as oscillations of response times as a function of the fixation-target interval (jitter interval). In fact, due to the jitter varying between 2-11 seconds, spectral decompositions of that signal were limited to resolving oscillations of 0.5 Hz or lower, which were out of the scope of our hypothesis (2-32 Hz). While rhythmicities in similar behavioral data have been validated in previous studies (Fiebelkorn et al., 2013, 2018; Helfrich et al., 2018), future studies using a jitter interval between 0.5 and 1.7 s could explore behavioral periodicities using methodologies similar to the ones used by Fiebelkorn et al. (2013). While consistent with frontocentral mechanisms of attention (Posner and Petersen, 1990; Fan et al., 2002, 2005; Busch et al., 2009; Busch and VanRullen, 2010; Chakravarthi and VanRullen, 2012; Dugué et al., 2015; McLelland et al., 2016), we only monitored at F3 and F4 with high-density bifrontal arrays. Full scalp montages could facilitate source reconstruction and otherwise test the spatial extent of attention-related signals (Michel and He, 2019). The results of the present study are also limited by the small cohort size that met our stringent exclusion criteria. While these exclusions improve confidence in our findings, future studies in larger cohorts could examine the generalizability of our results and support additional confound mitigation (Zoefel and Heil, 2013).

## 5. Conclusions

Our results suggest that alerting attention to perceptually suprathreshold stimuli, a fundamental aspect of cognition, is sufficient to elicit observable phase-dependent modulation of behavior that can be predicted by the phase of ongoing brain rhythms, particularly in the *θ* band in frontal regions of the brain. It is possible to identify these signals using high-density, dry EEG, at sub-second timescales in single subjects. Due to the absence of a pre-stimulus cue in our behavioral task, our findings indicate that internal, endogenously generated rhythms modulate the allocation of cognitive resources toward environmental stimuli. The behavioral relevance of multiple well-separated low-frequency rhythms suggests that different rhythms may serve different roles in the resource allocation process. The inter-subject variability of the neuronal rhythms whose phase predicts alertness suggests that brain rhythms might reflect differences in the overall efficiency of alertness across subjects.

This work has potential clinical relevance for neurological and psychiatric disorders that can be traced back to abnormal brain rhythms or can benefit from targeted neuromodulation during specific cycles of neuronal activity identified in single subjects (Zrenner et al., 2018, 2020). Closed-loop EEG-TMS interventions have been shown to produce favorable outcomes in patients suffering from disorders such as major depressive disorder and autism-spectrum disorder (Tremblay et al., 2019) and effective open-loop intervention protocols are rapidly emerging (Cole et al., 2020). Integrating those paradigms with non-invasive hardware that enables more accurate tracking of underlying brain rhythms and that can be customized to patient-specific anatomical and functional targets, could further improve therapeutic effectiveness and overall outcomes.

## Data availability statement

The raw data supporting the conclusions of this article will be made available by the authors, without undue reservation.

## Ethics statement

The studies involving human participants were reviewed and approved by Institutional Review Board of Drexel University (protocol no. 1904007140). The participants provided their written informed consent to participate in this study.

## Author contributions

Conceptualization: GM, BE, ND, JDM, and FV. Methodology: GM, BE, JDM, and FV. Software, validation, and data curation: GM and BE. Formal analysis: GM. Investigation: GM, BE, ND, SS, BK, RR, GF-N, and HS. Resources and funding acquisition: JDM and FV. Writing—original draft: GM, BE, and FV. Writing—review and editing: ND, SS, BK, RR, GF-N, and JDM. Visualization: GM, ND, and FV. Supervision and project administration: BE, JDM, and FV. All authors contributed to the article and approved the submitted version.
