# Supplementary material for: Alerting attention is sufficient to induce a phase-dependent behavior that can be predicted by frontal EEG
Source: Front Behav Neurosci. 2023 May 24;17:1176865. doi: 10.3389/fnbeh.2023.1176865 (PMC10246752; doi:10.3389/fnbeh.2023.1176865)
Supplement: Supplementary file 1 [file Data_Sheet_1.pdf]

Supplementary Material

SUPPLEMENTARY TABLES

Table S1. Number of trials after sequential preprocessing steps. **Valid** Number of valid trials before preprocessing, **NB** Number of valid trials after rejecting trials contaminated by blinks, **NB-NEMG** Number of valid trials after rejecting trials contaminated by blinks and EMG, **NB-NEMG-NRTO** Number of valid trials after rejecting trials contaminated by blinks, EMG, and response time outliers.

Participant	Valid	NB	NB-NEMG	NB-NEMG-NRTO
S1	176	85	79	77
S2	167	69	69	69
S3	182	72	71	70
S4	173	114	114	111
S5	176	73	73	71
S6	161	141	137	132
S7	172	119	98	96
S8	175	100	93	92
S9	177	86	81	79
S10	178	54	54	54
S11	173	71	70	69
S12	176	42	41	41
S13	181	58	58	57
S14	179	87	82	81
S15	174	58	53	52
mean $\pm$ SD	176 $\pm$ 5.3	78.6 $\pm$ 24.9	78.2 $\pm$ 24.8	76.7 $\pm$ 23.7

SUPPLEMENTARY FIGURES

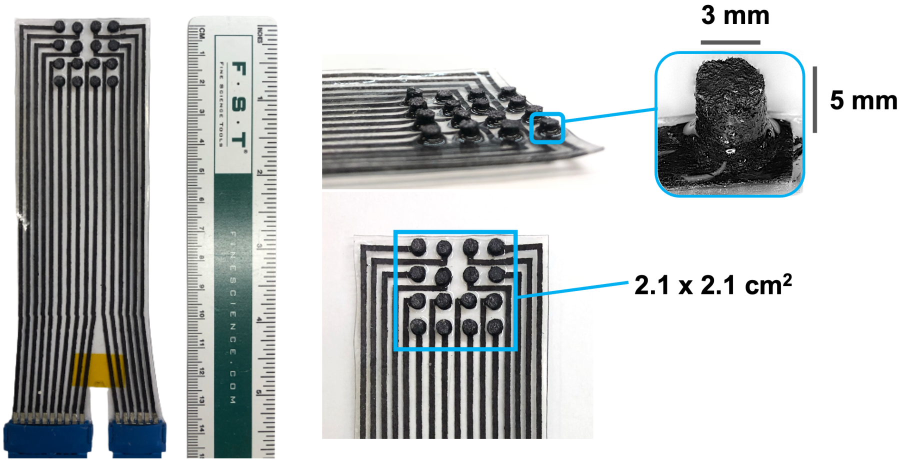

Figure S1: **High-density dry EEG arrays used in this study**. Each array is a 4x4 matrix of dry 3D electrodes (3 mm diameter, 5 mm height, 6 mm pitch each) covering an area of 21x21 mm^2. Here we depict (left) the full device, (right) side and top view of the array, with a close-up view of the 3D electrode contact.

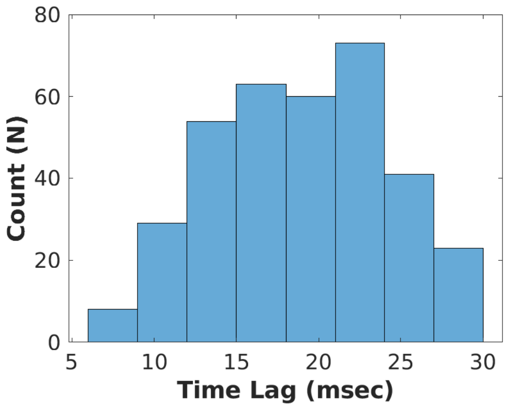

Figure S2: **Time lag between behavioral paradigm and EEG. Total number of trials n = 351.**

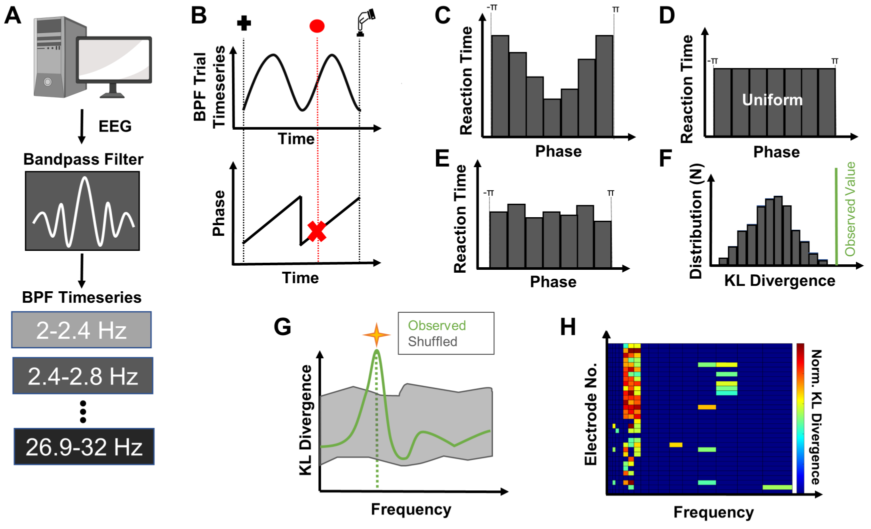

Figure S3: **Phase behavior analysis flow.** **(A)** Schematic of EEG data filtering into 17 logarithmically spaced frequencies (2-32 Hz). **(B)** Bandpass filtered EEG timeseries of one trial (top) and the associated phase (bottom). The red cross shows the phase at the time of target presentation. **(C)** Distribution of reaction times as a function of the phase angles ($\pm 45^o$) at the time of target presentation. **(D)** The uniform distribution against which we compared our observed distribution of response time vs phase (C) using KL divergence **(E)** Distribution of response times over phase after shuffling the response times across phase. **(F)** The surrogate distribution of KL divergence values obtained after shuffling the observed phase bin - response time pairs 1,000 times and each time computing the KL Divergence of each shuffled distribution against a uniform distribution. The observed value is shows as a green line. **(G)** KL divergence values obtained by comparing the observed distribution against the uniform distribution overlaid across the surrogate distribution of KL divergence values obtained after shuffling the observed response time phase pairs and comparing their distribution to the uniform distribution. **(H)** Colormap representing the normalized KL divergence obtained for each frequency band and electrode location.

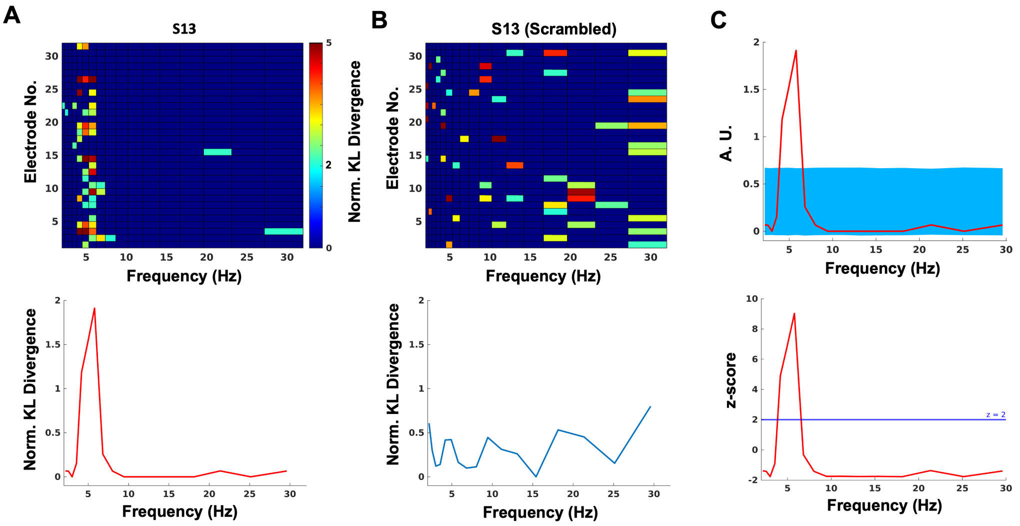

Figure S4: **The permutation test used to identify the frequency whose phase can significantly predict response times irrespective of electrode location for a representative participant.** **(A)** Top: colormap of KL divergence values across electrodes and frequencies. Bottom: averaged normalized KL divergence values in each frequency band across electrodes. **(B)** Top: colormap of KL divergence values whose phase can significantly predict response times after permuting across both the frequency and electrode location axes. Bottom: averaged normalized KL divergence values that were obtained in each frequency band across electrodes. **(C)** Top: observed average norm. KL divergence value (red), overlaid in the surrogate distribution ($\mu + 2\sigma$, baby blue) after 10,000 permutations. Bottom: z-score of the norm. KL divergence values of the observed distribution against the surrogate distribution at each frequency. Horizontal line is the significance threshold of $z > 2$ ($p < 0.05$).

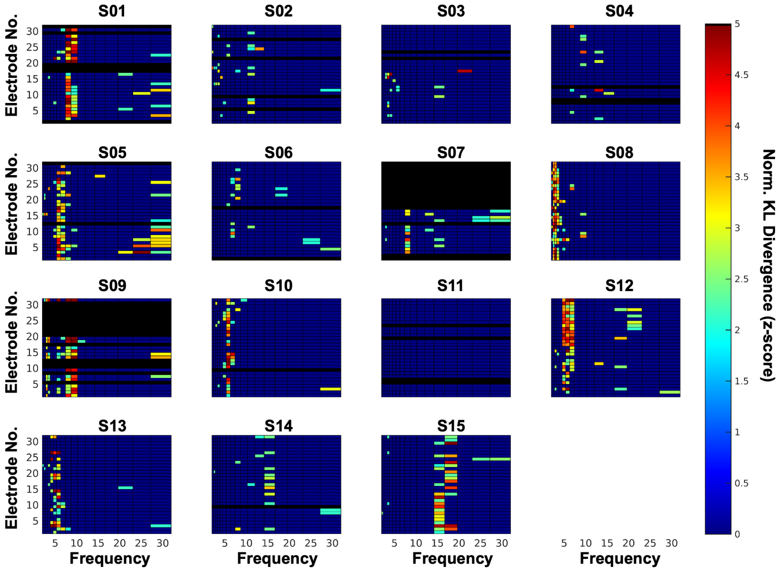

Figure S5: **Phase-behavior analysis results for each of the n=15 participants.** Panels show the subject-level colormap of the normalized KL divergence at each frequency band and electrode location (non-significant values were set to 0). Black rows in the subject-level colormaps denote high impedance channels.

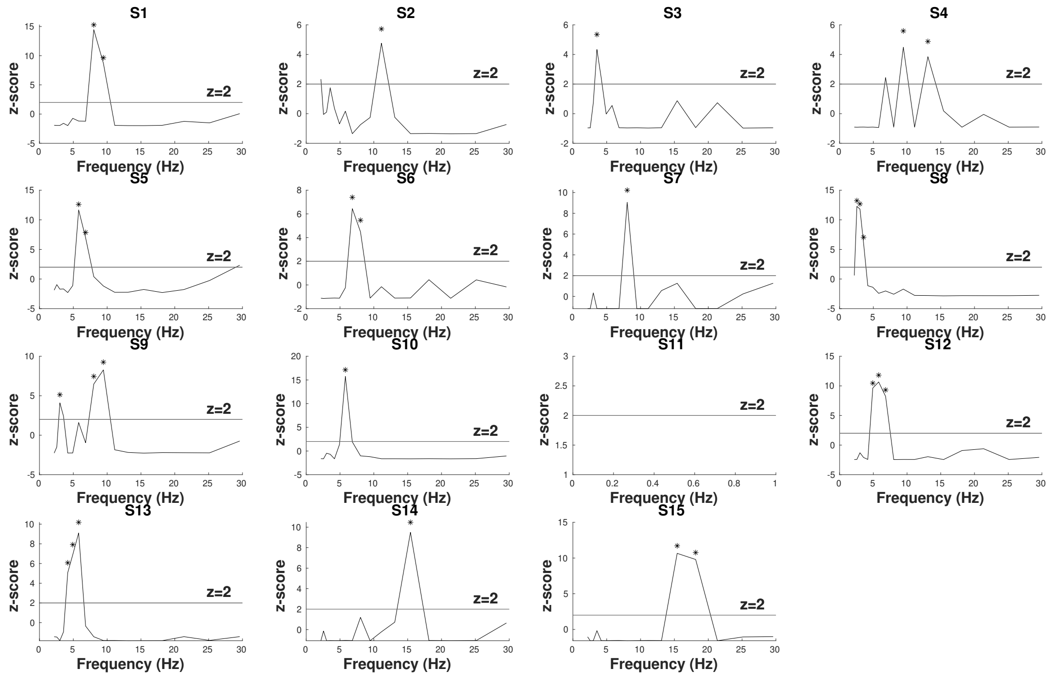

Figure S6: **Results of the permutation test to identify the frequency whose phase can predict participant response time irrespective of channel location.** Each panel shows the normalized KL divergence (z-score) averaged across all electrodes of the participant as a function of frequency. Values marked with an asterisk '*' represent significant values after correction for multiple comparisons.

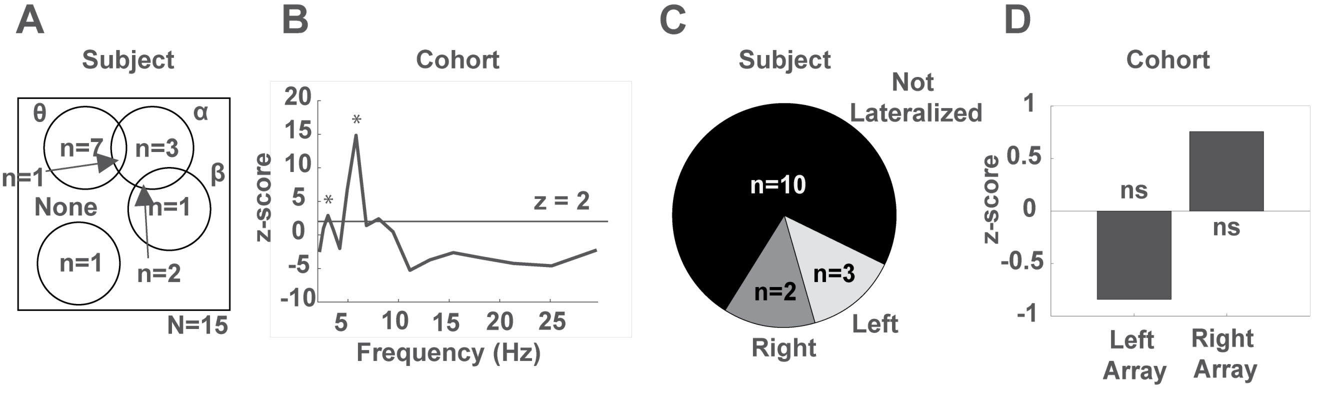

Figure S7: **Phase-behavior effects are strongest in the θ band across the frontal region when response times are not detrended as a function of trial number to account for fatigue. A**. Venn diagram of the phase-behavior distribution across frequency bands in our cohort. **B**. Z-scores obtained by comparing the observed distribution of response times over phase to the uniform distribution using a permutation test across our cohort. Values marked with an asterisk '*' represent significant values after correction for multiple comparisons. **C**. Distribution of the lateralization of phase-behavior effects within subjects. **D**. Lateralization effects at the cohort level. ns: not significant.

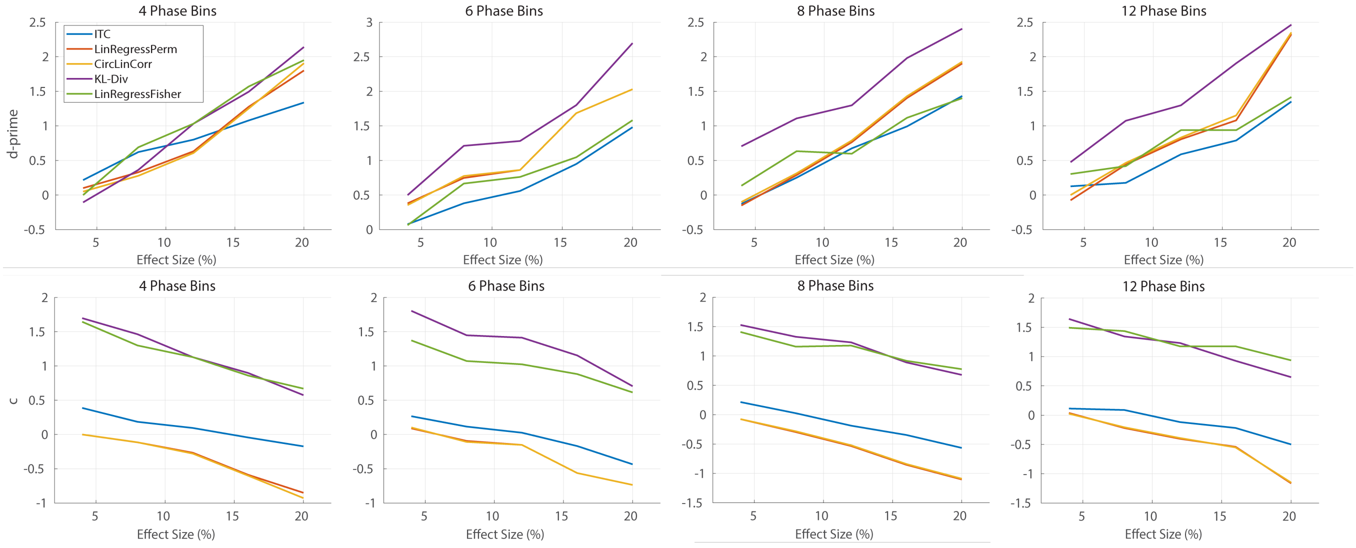

Figure S8: **Sensitivity (Top Row) and Bias (Bottom Row) of different methods commonly used to investigate phase behavior relationships and comparison of those with the method used in this study (KL-Div).** We used the code "simulate_all_methods_continuous_outcome.m" provided by Zoefel et al. (2019) available here using the default parameters *except: (1) no_subj = 1, effect_mu = 2.5, effect_sigma = 0.7, ampl = 4:4:20* to generate synthetic behavioral responses and their associated phase values for cohorts of size 1 where a phase-behavior effect was present or absent. We performed 500 tests where a phase-behavior effect was present at different levels (5 levels: 4, 8, 12, 16, 20 %, 100 tests per level) and 100 tests where a phase-behavior effect was absent. For each test, we quantified the ability of our statistical procedure using KL Divergence (KL-Div) to identify (or show absence of) any phase-behavior effects that were present. We compared its performance against 4 other commonly used approached based on: (1) linear regression followed by a permutation test (LinRegressPerm); (2) linear regression followed by Fisher's method for statistical significance (LinearRegressFisher); (3) inter-trial coherence (ITC); (4) circular to linear correlation (CircLinCorr), to detect any phase behavior relationships (or the lack thereof), which were pre-implemented by Zoefel et al. (2019).
